# Endovascular Management of Hemobilia as a Complication of Percutaneous Biliary Drainage

**DOI:** 10.7759/cureus.105708

**Published:** 2026-03-23

**Authors:** Alberto Latorre Pinto, Alberto Latorre Abisambra, Andres Latorre, Jose Brito

**Affiliations:** 1 Radiology, Hospital Universitario San Ignacio - Universidad Javeriana, Bogotá, COL; 2 Interventional Radiology, Cerid S.A., Barranquilla, COL; 3 Interventional Radiology, Universidad Autónoma de Bucaramanga, Bucaramanga, COL

**Keywords:** endovascular embolization treatment, hemobilia treatment, hepatic artery pseudoaneurysm, interventional radiology-guided embolization, percutaneous transhepatic biliary drainage

## Abstract

Hemobilia is an uncommon but potentially life-threatening cause of upper gastrointestinal bleeding, resulting from an abnormal communication between the biliary tract and adjacent vascular structures. In contemporary practice, iatrogenic injury has become the leading cause of hemobilia, particularly in the setting of invasive hepatobiliary procedures. Among these, percutaneous transhepatic biliary drainage is a well-recognized but infrequent source of vascular complications.

We report the case of a 74-year-old female patient with obstructive jaundice secondary to a hilar tumoral lesion who underwent percutaneous transhepatic biliary drainage. Approximately 36 hours after the procedure, she developed clinically significant hemobilia with hemodynamic compromise, manifested by active bleeding through the biliary drainage catheter and acute anemia requiring transfusion of two units of packed red blood cells. Cholangiography demonstrated a dilated biliary tree with intraductal filling defects consistent with blood clots. Subsequent selective hepatic arteriography revealed a hepatic arterial pseudoaneurysm with active contrast extravasation into a biliary radicle. The lesion was successfully treated with selective transarterial endovascular embolization, achieving complete exclusion of the pseudoaneurysm and resolution of the bleeding.

This case highlights the pathophysiological mechanisms of iatrogenic hemobilia following biliary interventions and underscores the importance of early recognition. It also emphasizes the pivotal role of angiography and transarterial embolization as first-line diagnostic and therapeutic tools in the management of this potentially severe complication.

## Introduction

Hemobilia is defined as the presence of hemorrhage within the biliary system secondary to an abnormal communication between the biliary tract and the adjacent vasculature. Although it represents an uncommon cause of upper gastrointestinal bleeding, its clinical relevance lies in the variability of its presentation, the associated diagnostic challenges, and the risk of potentially life-threatening hemorrhage. Historically, hemobilia was described in relation to hepatic artery aneurysms; however, its etiological profile has changed substantially in contemporary practice [1,2].

The classic Quincke triad - gastrointestinal bleeding, right upper quadrant pain, and jaundice - is considered characteristic of hemobilia, but it is present in only 22-35% of cases [3]. Consequently, diagnosis is often delayed, particularly when symptoms are intermittent or occur in a delayed fashion following a hepatobiliary procedure.

In current clinical practice, iatrogenic hemobilia represents the most frequent cause, accounting for more than half of reported cases. This increase is directly related to the growing number of diagnostic and therapeutic hepatobiliary procedures, including percutaneous, endoscopic, and surgical interventions. Reported iatrogenic causes include percutaneous or transjugular liver biopsy, transhepatic cholangiography, percutaneous transhepatic biliary drainage, transjugular intrahepatic portosystemic shunt creation, thermal ablation techniques, endoscopic retrograde cholangiopancreatography, biliary stent placement, and major hepatopancreatobiliary surgeries [2-4].

Within this spectrum, percutaneous transhepatic biliary drainage represents a particularly relevant cause. Although it is considered a safe and effective procedure for the management of biliary obstruction, hemorrhagic complications may occur as a result of direct arterial or venous injury, formation of vasculobiliary fistulas, or the development of arterial pseudoaneurysms. A distinctive feature of this entity is its delayed presentation, which is attributed to the initial tamponade effect exerted by the drainage catheter [4,5].

Multiphasic computed tomography is the initial imaging modality of choice in the acute setting; however, catheter-based angiography remains the reference standard, as it allows precise identification of the bleeding source and enables definitive treatment through endovascular techniques. Transarterial embolization has become the therapeutic strategy of choice in clinically significant cases, demonstrating high success rates and lower morbidity compared with surgical management [2,5].

## Case presentation

**Figure 1:**
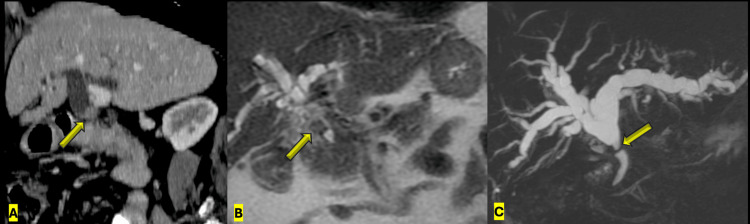
Cross-sectional imaging findings of proximal biliary obstruction A: Contrast-enhanced CT (oblique coronal reconstruction) demonstrating dilation of the common hepatic duct with an intraductal nodular soft-tissue lesion (arrow). B: Coronal T2-weighted MRI showing a hypointense nodular lesion at the confluence of the right and left hepatic ducts, with proximal intrahepatic biliary dilation and short-segment narrowing of the distal common hepatic duct. C: Oblique maximum intensity projection (MIP) reconstruction demonstrating intrahepatic ductal dilation up to the biliary confluence, short-segment occlusion, and visualization of the distal hepatocholedochus beyond the obstruction.

A 74-year-old female patient presented with right upper quadrant abdominal pain associated with progressive jaundice of the skin and sclera. Initial laboratory studies demonstrated a marked cholestatic pattern, with a total bilirubin level of 13.6 mg/dL, direct bilirubin of 9.3 mg/dL, and alkaline phosphatase of 915 U/L, accompanied by mild elevations in amylase and lipase. Coagulation parameters were within normal limits, including a normal international normalized ratio (INR) and platelet count, and the patient was not receiving anticoagulation therapy. Abdominal ultrasound revealed dilation of the intrahepatic biliary ducts. Subsequently, abdominal computed tomography and magnetic resonance cholangiography demonstrated dilation of the intrahepatic bile ducts secondary to an occlusive tumoral lesion adjacent to the confluence of the right and left hepatic ducts, findings consistent with proximal biliary obstruction of probable tumoral origin (Figure 1).

**Figure 2:**
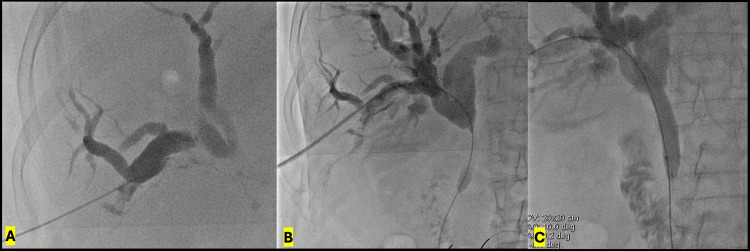
Percutaneous transhepatic cholangiography and biliary intervention A: Percutaneous cholangiography using a 21-gauge Chiba needle via a right peripheral biliary radicle, demonstrating diffuse intrahepatic biliary dilation. B: Hydrophilic 0.035-inch Roadrunner guidewire (Cook Medical, Bloomington, IN) traversing the occluded common hepatic duct, with the distal tip positioned in the proximal jejunum. C: Balloon dilation of the occluded segment using an 8.0 × 40 mm angioplasty balloon (Mustang; Boston Scientific, Marlborough, MA).

**Figure 3:**
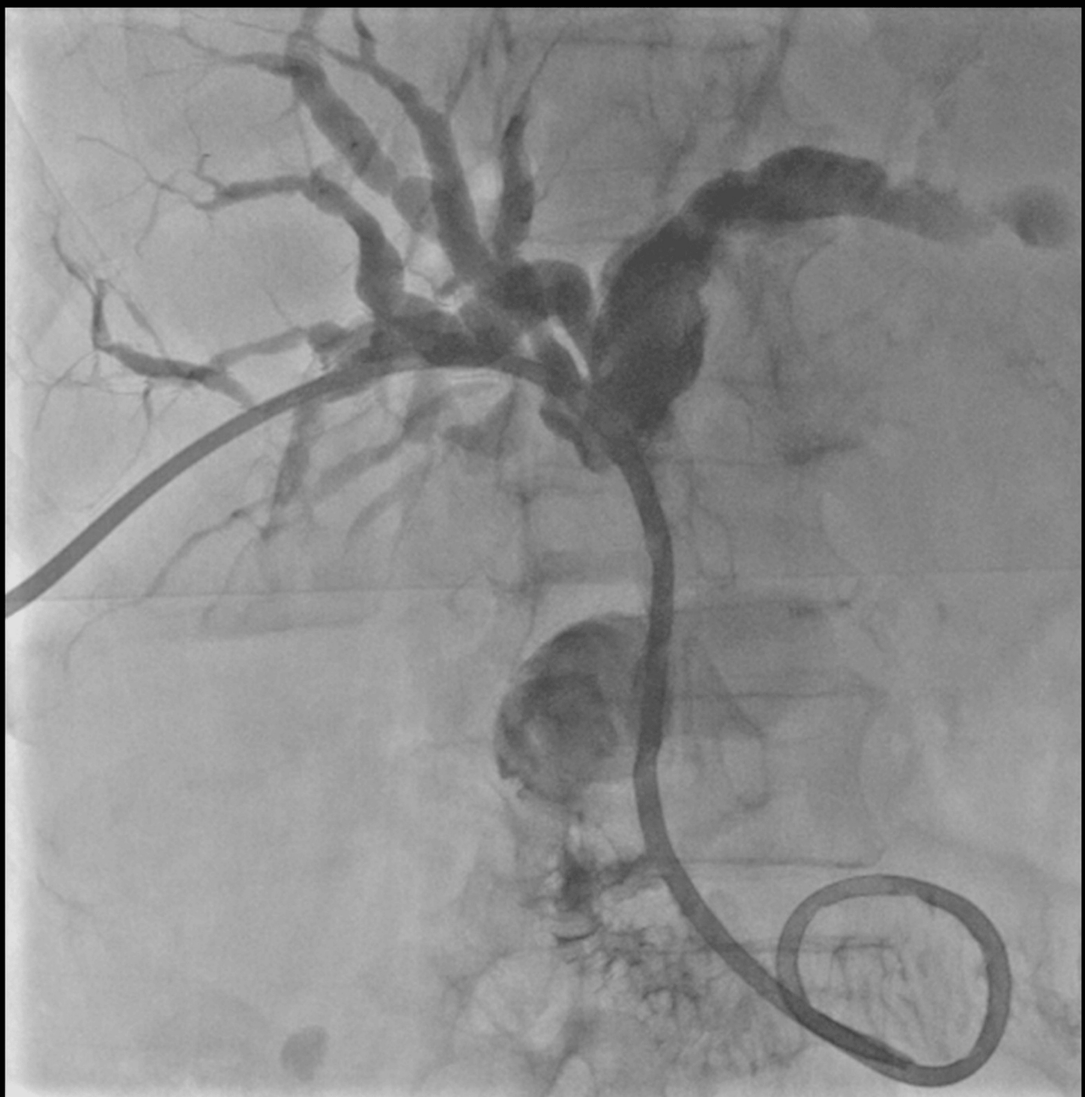
Immediate post-procedural control cholangiography Cholangiography performed through a 10-French internal-external biliary drainage catheter (Flexima Biliary; Boston Scientific, Marlborough, MA), demonstrating adequate opacification of the biliary tree without contrast extravasation or fistulous tracts. The distal catheter tip is positioned within the small bowel.

Based on these findings, an internal-external percutaneous transhepatic biliary drainage was performed via a right peripheral approach using a 21-gauge Chiba needle (Cook Medical, Bloomington, IN), which helps reduce the risk of vascular injury-related complications. During the procedure, dilation of the intrahepatic biliary tree up to the confluence was identified, with a short-segment occlusion of the common hepatic duct. The lesion was successfully crossed using a hydrophilic guidewire (Roadrunner 0.035 × 260 cm) supported by a 5-French multipurpose catheter. Balloon dilation of the common hepatic duct and common bile duct up to the ampulla was performed using an 8 × 40 mm balloon, followed by placement of a 10-French internal-external biliary drainage catheter with side holes proximal and distal to the lesion. No contrast extravasation or other abnormalities were observed on control cholangiography through the catheter (Figures 2-3). The patient tolerated the procedure well and was transferred to the inpatient ward for observation.

**Figure 4:**
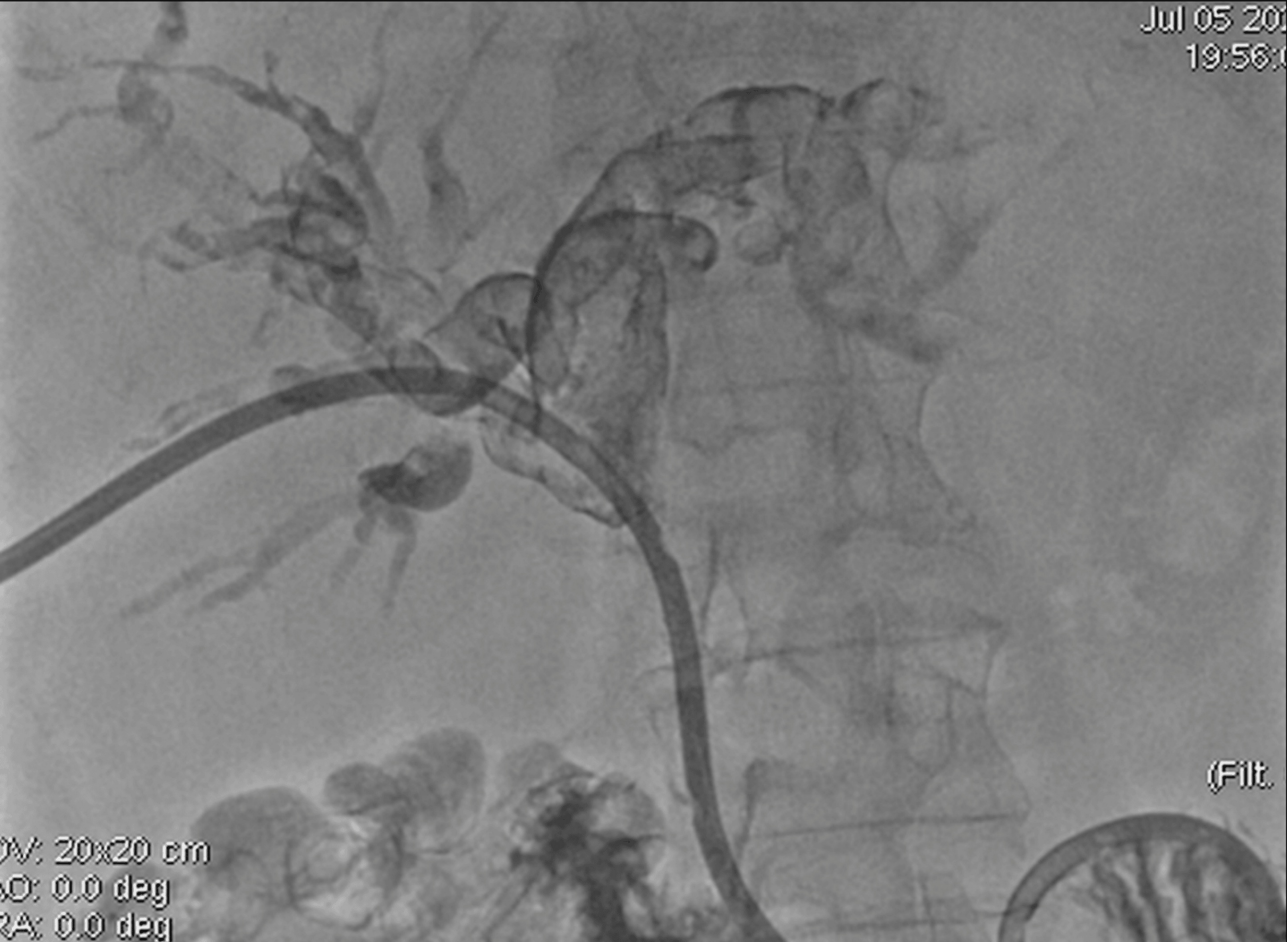
Cholangiographic findings of hemobilia Cholangiography through the biliary drainage catheter, demonstrating multiple linear and rounded intraductal filling defects, consistent with intrabiliary clot formation in the setting of hemobilia.

**Figure 5:**
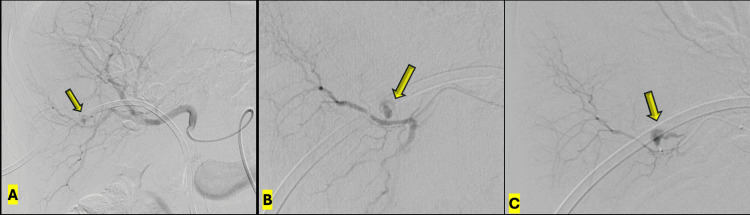
Angiographic identification of the hepatic arterial pseudoaneurysm A: Hepatic arteriography using a 5-French Cobra catheter (Cordis, Miami Lakes, FL) in the proper hepatic artery, demonstrating a small pseudoaneurysm (arrow) arising from an intrahepatic branch supplying the posteroinferior right hepatic lobe. B: Early-phase superselective arteriography using a PX Slim microcatheter (Penumbra, Alameda, CA) over a PT2 microguidewire, demonstrating a small pseudoaneurysm, with contrast outlining the side hole of the biliary drainage catheter (arrow) and tracking into the biliary system, consistent with active hemobilia. C: Late-phase superselective arteriography demonstrating complete opacification of the pseudoaneurysm with contrast passage into the adjacent biliary radicle (arrow).

**Figure 6:**
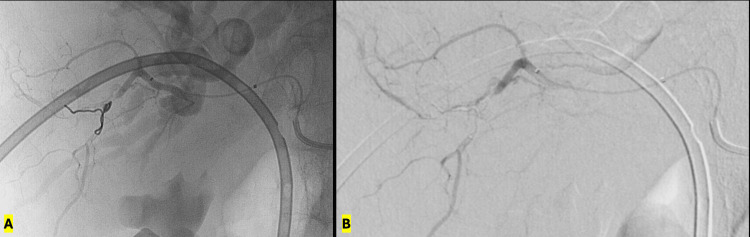
Endovascular embolization and angiographic outcome A: Deployment of 2 mm × 4 cm Ruby Soft microcoils (Penumbra Inc., Alameda, CA) achieving occlusion of the pseudoaneurysm. B: Post-embolization superselective arteriography demonstrating complete exclusion of the pseudoaneurysm with preserved patency of adjacent hepatic arterial branches.

Approximately 36 hours after biliary drainage, the patient developed active bleeding through the biliary drainage catheter, with a significant decline in hemoglobin levels, requiring transfusion of two units of packed red blood cells, consistent with hemobilia. Cholangiography performed through the catheter demonstrated filling defects compatible with intraductal blood clots (Figure 4), followed by selective hepatic arteriography, which revealed a saccular pseudoaneurysm arising from a segmental branch of the right hepatic artery, with active contrast extravasation and communication with a biliary radicle (Figure 5). Selective endovascular embolization using microcoils was performed, achieving complete exclusion of the pseudoaneurysm and cessation of bleeding (Figure 6). The subsequent clinical course was favorable, with no recurrence of hemobilia. Six weeks later, after maturation of the tract, a percutaneous biopsy of the biliary lesion was performed using the SpyGlass™ Discovery system (Boston Scientific, Marlborough, MA), without additional hemorrhagic complications.

## Discussion

Iatrogenic hemobilia represents an uncommon but clinically significant complication of hepatobiliary procedures. Its pathophysiology is based on the creation of an abnormal communication between the vascular system and the biliary tract, either due to direct injury during puncture, formation of vasculobiliary fistulas, or development of arterial pseudoaneurysms that erode into the bile ducts [2].

Percutaneous transhepatic biliary drainage is one of the most frequently implicated iatrogenic causes. The incidence of bleeding following this procedure has been reported to range between 2% and 5%, with clinically significant hemobilia being less frequent [4]. Although the overall incidence of post-procedural bleeding is low, hemobilia may present in a delayed fashion due to the initial tamponade effect exerted by the drainage catheter. When bleeding occurs, it may manifest as gastrointestinal hemorrhage, obstructive jaundice secondary to intraductal clots, or bleeding through the drainage catheter, as observed in the present case [4-6].

Previous studies have described that vascular complications associated with percutaneous transhepatic biliary drainage may be related to both technical factors - such as puncture trajectory, number of needle passes, and proximity to arterial branches - as well as patient-related factors, including coagulation abnormalities and local inflammatory changes. In particular, arterial pseudoaneurysms represent one of the most relevant complications due to their potential for massive bleeding and delayed presentation, findings that are consistent with the case presented. In the present case, angiography demonstrated a hepatic arterial pseudoaneurysm located adjacent to the side hole of the biliary drainage catheter, suggesting a direct communication between the arterial injury and the drainage tract, which explains the delayed hemobilia observed. The literature emphasizes that angiography not only allows confirmation of the diagnosis but also constitutes the therapeutic approach of choice, positioning endovascular embolization as the first-line treatment with high success rates and low morbidity [2,5,6].

From a diagnostic standpoint, multiphasic computed tomography allows identification of intraductal blood, pseudoaneurysms, and active extravasation; however, catheter-based angiography provides precise characterization of the vascular lesion while simultaneously enabling immediate therapeutic intervention. Once the bleeding source is identified angiographically, management is directly oriented toward selective endovascular exclusion of the injured vessel, an approach that has demonstrated high technical and clinical success rates with a favorable safety profile [2,5].

The management of hemobilia secondary to visceral arterial pseudoaneurysms following percutaneous transhepatic biliary drainage relies on targeted endovascular therapy, which has shown high success rates and has relegated surgery to a rescue role [4,5]. Selection of the embolic agent should be individualized according to pseudoaneurysm morphology, arterial caliber, the presence of a wide neck, and the need to preserve hepatic perfusion. Metallic coils are highly effective in narrow-neck pseudoaneurysms by inducing mechanical occlusion and controlled thrombosis [5], whereas liquid embolic agents, such as n-butyl cyanoacrylate (NBCA) or ethylene vinyl alcohol (EVOH) copolymers, allow more complete exclusion in complex or wide-neck lesions, albeit with higher technical demands [7]. Recent evidence suggests that combined strategies using coils and liquid embolic agents may reduce recanalization rates in complex visceral pseudoaneurysms [8], consolidating endovascular embolization as the treatment of choice [4].

This case reinforces the need to maintain a high index of clinical suspicion in the presence of unexplained bleeding or changes in biliary drainage output, particularly during the early or late post-procedural period. Timely recognition and appropriate endovascular management are essential to reduce the morbidity and mortality associated with this entity.

## Conclusions

Iatrogenic hemobilia secondary to percutaneous transhepatic biliary drainage is an uncommon but potentially severe complication that forms part of a broader spectrum of vascular complications described after this type of intervention. Its clinical presentation may be variable and delayed, requiring a high degree of diagnostic suspicion. Catheter-based angiography plays a central role by allowing precise identification of the bleeding source and definitive treatment through transarterial embolization. This case specifically illustrates a hepatic arterial pseudoaneurysm in direct relation to the side hole of the biliary drainage catheter as a mechanism of delayed hemobilia. The present case aligns with reports in the literature regarding delayed vascular complications and highlights the efficacy and safety of endovascular management as a first-line therapeutic approach. A timely and multidisciplinary strategy is essential to optimize clinical outcomes.
